# A study on matching supply and demand of ecosystem services in the Hexi region of China based on multi-source data

**DOI:** 10.1038/s41598-024-51805-1

**Published:** 2024-01-16

**Authors:** Xuebin Zhang, Xuehong Li, Ziyang Wang, Yue Liu, Litang Yao

**Affiliations:** 1 College of Geographic and Environmental Science, Northwest Normal University, Lanzhou, Gansu Province China; 2 Key Laboratory of Resource Environment and Sustainable Development of Oasis, Lanzhou, Gansu Province China; 3 Gansu Engineering Research Center of Land Utilization and Comprehension Consolidation, Lanzhou, China

**Keywords:** Ecology, Environmental sciences

## Abstract

To achieve the best management of the ecosystem and sustainable socioeconomic development, it is crucial to clarify the matching relationship between the supply and demand of ecosystem services (ESs). Four types of ESs were chosen for the Hexi region in this study: food supply, carbon sequestration, water yield, wind erosion control and sediment retention. We assessed the supply–demand and matching relationships of different ESs using the Integrated Valuation of Ecosystem Service and Tradeoffs (InVEST) model, the ESs supply–demand ratio and the four-quadrant model. Moreover, we also analyzed the supply–demand ratio of integrated ESs and their cold spots. The findings suggest that (1) from 2000 to 2020, the average supply of food supply, carbon sequestration, water yield, wind erosion control and sediment retention increased by 44.31 t/km^2^, 128.44 t/hm^2^, 14,545.94 m^3^/km^2^ and 0.14 kg/m^2^ respectively, which showed a spatial pattern of “high in the southeast and low in the northwest”. The average demand for food supply and carbon sequestration increased by 1.33 t/km^2^ and 0.32 t/hm^2^ respectively, while the average demand for water yield and wind erosion control and sediment retention decreased by 2997.25 m^3^/km^2^ and 1.19 kg/km^2^ respectively. The spatial distribution is consistent with the layout of population density, production and residential areas, and fragile ecological areas. (2) The supply–demand ratio of food supply, carbon sequestration and water yield is greater than 0.095, which is in a state of oversupply, and the supply–demand ratio of wind erosion control and sediment retention is less than 0, which is in a state of shortage; all ESs are mainly in low-low spatial matching areas, mainly concentrated in the desert areas of the northwest in the Hexi region. (3) The supply–demand ratio of integrated ESs increased by 0.024, and the proportion of cold spots and sub-cold spots was more than 50% and concentrated in the northwest, while hot spots and sub-hot spots accounted for only about 16%, mainly distributed in the southern Qilian Mountains and some oasis areas.

## Introduction

Ecosystem services (ESs) refer to all the benefits that human beings obtain directly or indirectly from the ecosystem^1^, including provisioning, regulation, culture and support services^2^, and they are fundamental to the survival and reproduction of life on Earth^3^. In addition, ESs also play a key supporting role in the security of the World Economic Forum (WEF)^4^. Since the Anthropocene, human activities have been increasingly interfering with ecosystems, resulting in a decline in ESs^5^, and posing great challenges to human well-being. For example, more than 60% of the world's ecosystems have been significantly degraded due to the expansion of construction land and rapid population growth^6–8^. Meanwhile, consumption demand is also increasing, which leads to the imbalance between supply and demand for ESs and a series of ecological and environmental problems. The phenomenon of the imbalance between supply and demand of ESs is rapidly spreading in many countries and regions, and has become the focus of academic attention^9–11^.

In recent decades, rapid socio-economic development and irrational human activities have led to ecosystem degradation^12^, which has greatly affected the balance between supply and demand of ESs and sustainable development^13^. To ensure the healthy and sustainable development of ecosystems and humans, the United Nations has included “protecting, restoring and promoting sustainable use of terrestrial ecosystems, sustainably managing forests, combating desertification, halting and reversing land degradation, and halting the loss of biodiversity” as one of its global sustainable development goals in “Transforming Our World: The 2030 Agenda for Sustainable Development”^14^. What’s more, the report of the 19th National Congress also proposed that “-it is necessary to provide more high-quality ecological products to meet the growing needs of the people for a beautiful ecological environment”^15^. Essentially, this reflects the problem of trying to match supply and demand. The Hexi region is an important part of the “Qinghai-Tibet Plateau Ecological Barrier” and the “Northern Sand Control Belt” in the “Two Screens and Three Zones” overall ecological security strategic pattern set out in the National Major Functional Area Plan. It is an important ecological security barrier in northwest China and even the whole country, performing important ESs and providing the region with ESs such as food supply, carbon sequestration, water yield, wind erosion control and sediment retention. Since the mid-1980s, with the rapid economic development, population growth and exploitation of soil and water resources, the ecological problems in the Hexi region have become more obvious, affecting the balance between supply and demand of ESs in the region. Therefore, it is urgent to clarify the supply and demand of ESs and their matching relationship.

Research on the supply and demand of ESs began with the study of ecological carrying capacity and the value of ESs in the 1990s^1,16^. The change in ESs supply and demand can reflect the dynamic process between the natural ecosystem and the human social system, which has become the hot topic of academic attention^9–11^. Since 2000, research on ESs supply and demand has mainly focused on basic and applied research, including conceptual discussion^17^, quantitative analysis on ESs supply and demand^18,19^, ESs types and spatial–temporal evolution^20,21^, reversibility trade-offs and synergies^11,22^, and the application of their research results^23^. The analysis of the characteristics of temporal and spatial variation and the matching relationship between supply and demand of specific ESs will help to comprehensively reveal the actual situation of each ESs and provide reference for ecosystem management. However, due to the incomplete development of theories, limited data collection and immature research methods, most of them focus on the comprehensive research of supplying, regulating, supporting and cultural services, and pay less attention to specific ESs.

With the development of technology, research methods on the supply and demand of ESs are gradually diversifying. Research methods include expert assessment of supply and demand matrices^24^, model calculation methods^22,25^, ecological process simulation^26^, data spatial overlay method, land use estimation, and value equivalents^7,27^. Some scholars have used population density, GDP per capita and land use intensity to assess the demand for ESs^7,27^. However, such methods did not take into account the specific needs of different ESs, which may lead to low accuracy in the assessment results. Therefore, it is necessary to use the actual physical quality of specific ESs to measure their demand, in order to clarify the specific needs of each ESs.

This study is based on multi-source data, selecting four types of ESs: food supply, carbon sequestration, water yield, and wind erosion control and sediment retention. The temporal and spatial evolution characteristics of the supply and demand of each ESs are analyzed using the InVEST model. The supply–demand ratios and the four-quadrant model are used to evaluate the supply–demand matching of each ESs. The comprehensive supply–demand ratio and its hot and cold spots are also analyzed. The objectives of the study are as follows: (1) To clarify the temporal and spatial characteristics of four ESs, namely food supply, carbon sequestration, water yield, wind erosion control and sediment retention. (2) To explore the supply–demand matching relationship of each ESs from both quantitative and spatial aspects. (3) To investigates the comprehensive supply–demand ratio of ESs and their hotspots and coldspots.

## Materials and methods

### Research framework

The supply and demand of ESs has become a bridge connecting natural systems and human social systems^28–30^, and it is of great significance in the process of achieving ecological security and sustainable social and economic development^31^. The Millennium Ecosystem Services Assessment (MA) classifies ESs into four categories: support services, provisioning services, regulating services and cultural services^2^. According to the actual situation of previous studies and research areas^2,15,29^, we selected two kinds of supply services: food supply and water yield, and two kinds of adjustment services: carbon sequestration and wind erosion control and sediment retention. Among them, food supply is not only the material basis of local residents' lives, but also an important support service. The food supply in the Hexi area is very important for the food security of Northwest China and even the country. Carbon sequestration is an important regulating service, which can increase the carbon absorption and storage capacity of the ecosystem, thus slowing down the increase in atmospheric CO^2^ concentration, slowing down global warming and maintaining the global carbon balance. The Hexi region is a severely water-poor region in China, and water production is an important water supply service for human production and life, which is very important for the sustainable development of the region. At the same time, the Hexi region is an important part of the “Northern Sand Prevention Belt” in the national ecological security strategic pattern of “Two Screens and Three Belts”. As one of the important regulating services, wind erosion control and sediment retention plays an important role in China’s ecological security and biodiversity protection. Therefore, it is very important to analyze the matching relationship between supply and demand of ESs from four aspects: food supply, carbon sequestration, water yield, wind erosion control and sediment retention.

**Figure 1:**
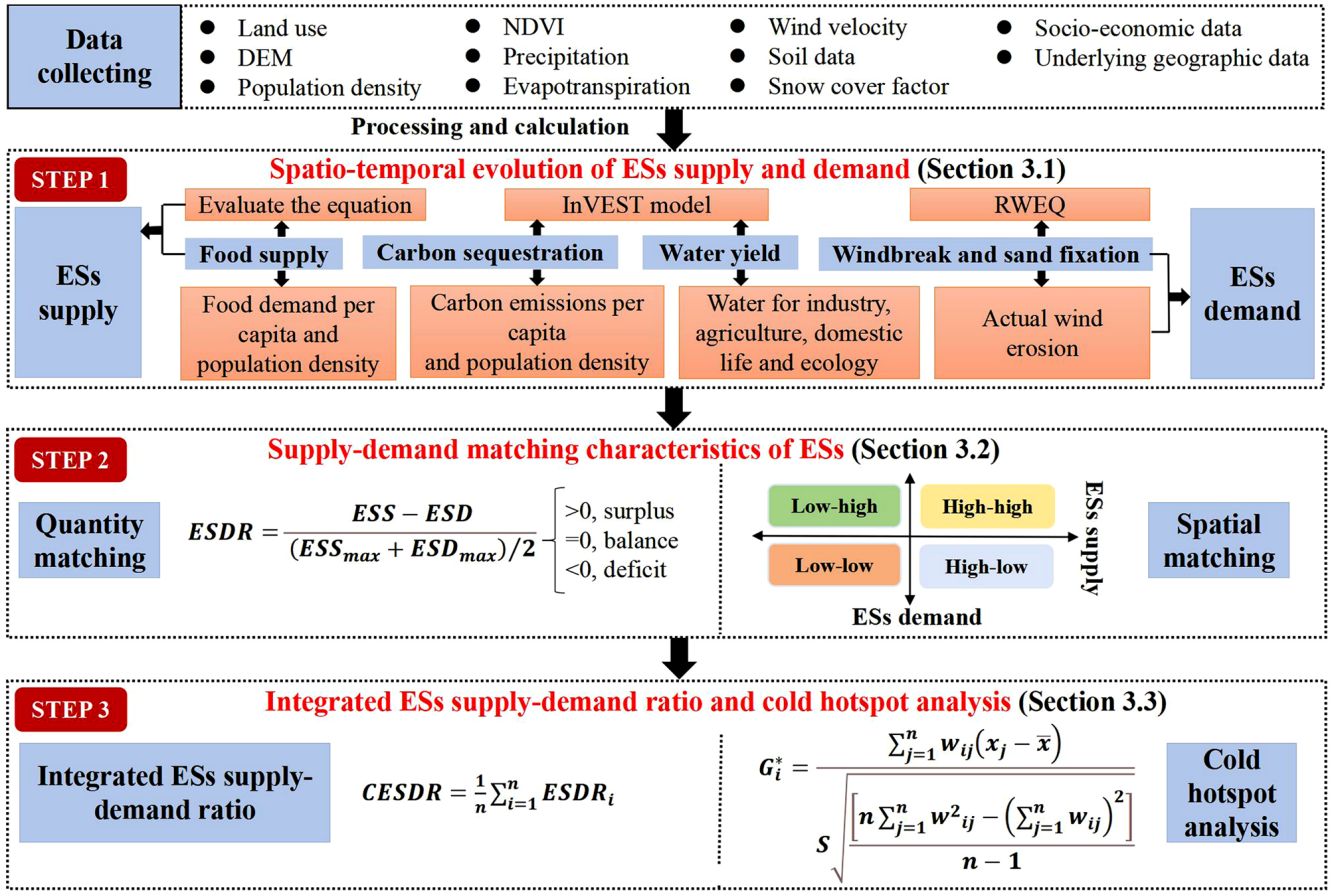
Research framework.

These four ESs influence and interact with each other. For example, the wind erosion control and sediment retention is the foundation that ensures the region is not affected by sandstorms, thus maintaining the sustainability of the other three ESs. The water yield can ensure the water demand for food production. The food supply and water yield can promote the carbon storage capacity of the ecosystem, and the carbon sequestration can regulate the ecosystem and have a positive impact on the water yield and food supply service^11,23,25,33^. However, rapid urbanization and irrational human activities have led to ecosystem degradation^12^, putting great pressure on the balance between supply and demand of ESs. Therefore, we constructed a framework to assess the matching of supply and demand of each ESs for regional sustainable development (Fig. 1).

### Study area

**Figure 2:**
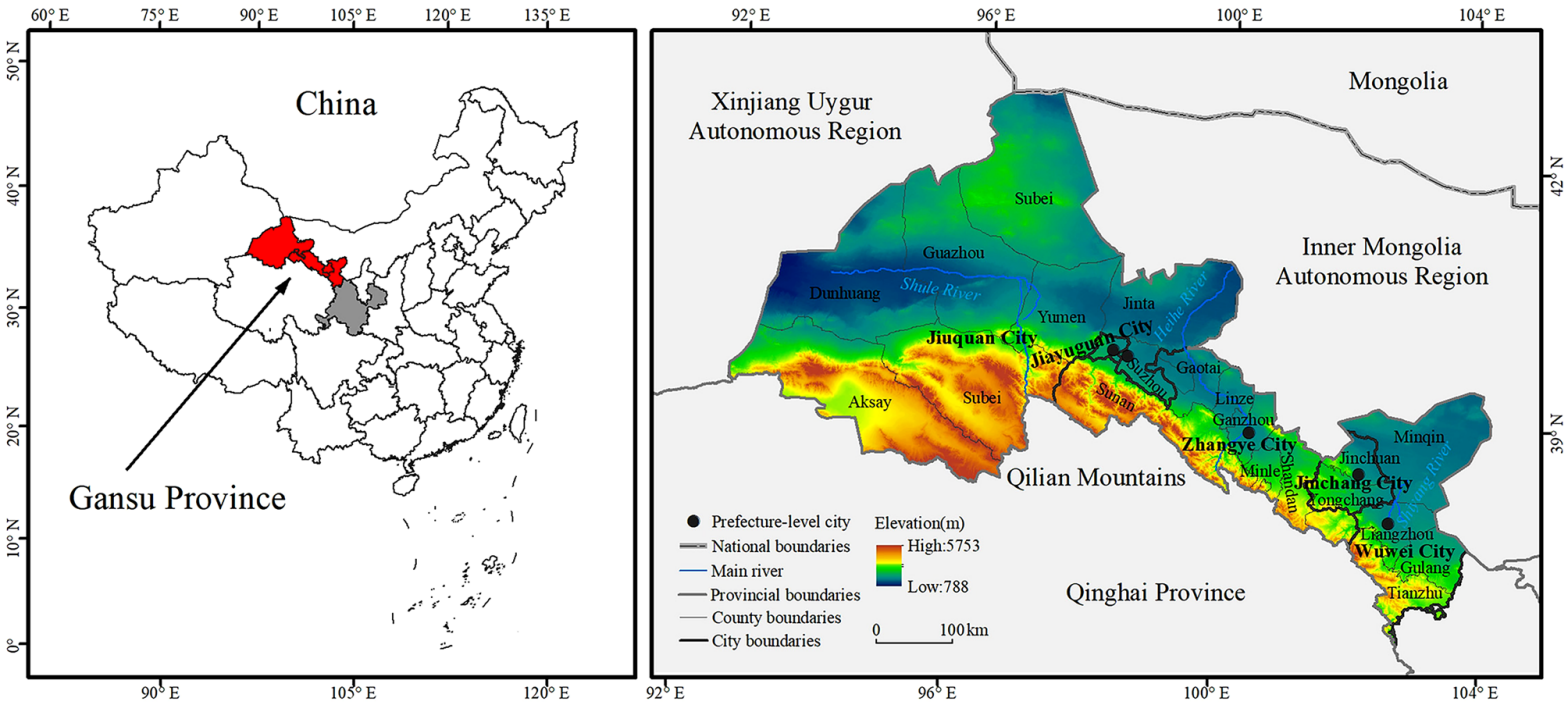
Study area. (The map was generated by ArcGIS 10.8 http://www.esri.com/software/arcgis and does not require any permission from anywhere).

The Hexi region is located at 37°17′–42°48′ N, 92°12′–103°48′ E in the northwest of Gansu Province, west of the Yellow River, and in the middle of the Qilian Mountains and the Badain Jaran Desert. It administratively covers five cities (Jiuquan, Jiayuguan, Zhangye, Jinchang, and Wuwei) and 20 counties/districts, with an east–west length of about 1000 km and a north–south width of nearly 100–200 km, and a total area of 254,200 km^2^ (Fig. 2). It is an important transportation hub connecting Central Asia, West Asia, and Europe. There are three rivers in the region, namely, the Shiyang River, Heihe River and Shule River, all of which originate in the Qilian Mountain glacier. At the same time, the region has formed a large area of oases due to the water resources provided by those rivers. The region belongs to the typical temperate continental climate, with an average annual precipitation of about 200 mm, and an average yearly temperature of 5.8–9.3 ℃. The evaporation is strong, and the wind and sand are frequent. The ecological environment is very fragile. By the end of 2020, the regional permanent population was 4.4024 million, and the urban population was 2.5798 million, with an urbanization rate reaching 58.6%. The GDP was 229.143 billion yuan, and the per capita GDP was 52,000 yuan. The economic development and rapid urbanization have leaded to the regional development. Still, at the same time, they have interfered with the ecological environment and aggravated the disharmony between human activities and geographical setting.

### Data source

The data used in this study are shown in Table 1. The grid size is unified to 1000 m by resampling, and the geographic coordinate system uses WGS_1984_Albers.

**Table 1:** Date sources and description.

Data	Data description	Sources	Resolution
Land use data	Raster data for 2000, 2010 and 2020	Data Center for Resources and Environmental Sciences, Chinese Academy of Sciences (http://www.resdc.cn)	30 m
DEM	Raster data, used for subbasin extraction and calculation of terrain factors		30 m
Population density	Raster data for 2000, 2010 and 2020		1 km
Basic geographic data	Vector data, including administrative boundaries and rivers		
NDVI	Raster data for 2000, 2010 and 2020	United States Geological Survey (USGS)	250 m
Rainfall and evapotranspiration	Raster data for 2000, 2010 and 2020	National Earth System Science Data Center(http://www.geodata.cn)	1 km
Wind speed, rainfall site data	The rainfall days, the average monthly wind speed of each month, and the number of days with a daily average wind speed greater than 5 m/s at 2 m in each month were obtained through station interpolation	National Meteorological Science Data Center (http://data.cma.cn/)	1 km
Soil data	Sand, Clay, Silt, Percentage of organic matter content, Calcium carbonate content	Harmonized World Soil Database (HWSD) (V1.2)	1 km
Snow cover factor	Raster data for 2000, 2010 and 2020	National Cryosphere Desert Data Center (http://www.ncdc.ac.cn/portal/)	1 km
Statistical data	Food production per capita, food demand per capita, water consumption and energy consumption for 2000, 2010 and 2020	China Statistical Yearbook, Gansu Development Yearbook, Gansu Rural Yearbook, Gansu Provincial Water Resources Bulletin, Statistical yearbooks of prefecture-level cities	–

### Assessment of supply and demand for ESs

The supply and demand for ESs are mainly assessed according to the formulas in the table below:

**Table:** 

Type	Calculation method	Calculation formula	Variable interpretation
Food supply	According to the ratio of ${{\text{NDVI}}}_{{\text{i}}}$ to ${{\text{NDVI}}}_{{\text{sum}}}$, the total food output in Hexi region was distributed by grid units, so as to determine the food supply of grid units^34^	${\text{G}}_{{\text{i}}} = {\text{G}}_{{{\text{sum}}}} \times \frac{{{\text{NDVI}}_{{\text{i}}} }}{{{\text{NDVI}}_{{{\text{sum}}}} }}{ }$ (1)	${\text{G}}_{{\text{i}}} { }$ indicates food supply of grid ${\text{ i}}$(t), ${\text{NDVI}}_{{\text{i}}} { }$ is the NDVI value of grid ${\text{ i }}$, and ${\text{NDVI}}_{{{\text{sum}}}}$ is the whole NDVI value of the study area,${\text{ G}}_{{{\text{sum}}}} { }$ represents the total food production (t)
	The food supply demand was estimated based on the food consumption per capita and population density^35^	${\text{D}}_{{\text{i}}} = {\text{D}}_{{{\text{per}}}} \times {\text{P}}_{{{\text{ipop}}}} { }$ (2)	${\text{D}}_{{\text{i }}}$ is the food supply demand of grid ${\text{i}}$(t),${\text{ D}}_{{{\text{per}}}} { }$ denotes the food consumption per capita (t/person),${\text{ P}}_{{\text{ipop }}}$ is the population Density (person/km^2^)
Carbon sequestration	The InVEST model carbon sequestration module was used to calculate the carbon sequestration supply^36^	$$\begin{aligned} {\text{S}}_{{{\text{cs}}}} & = {\text{C}}_{{{\text{above}}}} + {\text{C}}_{{{\text{below}}}} \\ & \;\;\; + {\text{C}}_{{{\text{soil}}}} + {\text{C}}_{{{\text{dead}}}} { } \\ \end{aligned}$$ (3)	${\text{S}}_{{{\text{cs}}}} { }$ is the carbon sequestration supply,${\text{ C}}_{{{\text{above}}}}$, ${\text{C}}_{{{\text{below}}}}$,${\text{ C}}_{{{\text{soil}}}}$,${\text{ C}}_{{{\text{dead}}}}$ are abovementioned biological carbon, underground biological carbon, soil organic carbon and dead organic matter (t/hm^2^), respectively
	The carbon sequestration demand was estimated based on the carbon emissions per capita and population density	${\text{D}}_{{{\text{cs}}}} = {\text{D}}_{{{\text{pcfc}}}} \times {\text{D}}_{{{\text{pop}}}} { }$ (4)	${\text{D}}_{{{\text{cs}}}} { }$ is the carbon sequestration demand,${\text{ D}}_{{{\text{pcfc}}}}$ represents the carbon emissions per capita(t/person),${\text{ D}}_{{{\text{pop}}}} { }$ is the population Density (person/km^2^)
Water yield	The InVEST model was used to quantify the water yield supply in Hexi Region^27^	${\text{W}}_{{{\text{xj}}}} = \left( {1 - \frac{{{\text{AET}}_{{{\text{xj}}}} }}{{{\text{P}}_{{\text{x}}} }}} \right) \times {\text{P}}_{{\text{x}}} { }$ (5)	${\text{W}}_{{{\text{xj}}}} { }$ represents the annual water yield supply (mm), ${\text{AET}}_{{{\text{xj}}}} { }$ is the annual evapotranspiration of type ${\text{ j }}$ land use and grid ${\text{ x}}$(mm),${\text{ P}}_{{\text{x }}}$ is the annual precipitation (mm)
	We distribute industrial water consumption equally to construction land; Agricultural water consumption is evenly distributed to cultivated land; Domestic water consumption is equally allocated to urban land and rural residential land; Ecological water consumption is evenly distributed to Forest land and grassland^37^	$$\begin{aligned} {\text{D}}_{{\text{Y}}} & = {\text{D}}_{{{\text{agricultural}}}} + {\text{D}}_{{{\text{industrial}}}} \\ & \;\;\; + {\text{D}}_{{{\text{domestic}}}} + {\text{D}}_{{{\text{ecological}}}} \\ \end{aligned}$$ (6)	${\text{D}}_{{\text{Y}}} { }$ is the water yield demand, ${\text{D}}_{{{\text{agricultural}}}}$,${\text{ D}}_{{{\text{industrial}}}}$, ${\text{D}}_{{{\text{domestic}}}} { }$ and ${\text{ D}}_{{\text{ecological }}}$ are respectively agricultural water consumption, industrial water consumption, domestic water consumption and ecological water consumption (m^3^)
Wind erosion control and sediment retention	Wind erosion control and sediment retention supply are calculated based on the difference between the amount of soil erosion in bare ground and vegetation protection. The Modified Wind Erosion Equation (RWEQ) was used to evaluate the supply of wind erosion control and sediment retention^32^	${\text{F}}_{{\text{S}}} = {\text{SL}}_{{\text{p}}} - {\text{SL}}_{{\text{r}}} { }$ (7) ${\text{SL}}_{{\text{p}}} = \frac{{2{\text{Z}}}}{{{\text{sp}}^{2} }} \times {\text{Q}}_{{\text{p}}} \times {\text{e}}^{{ - \left( {{\text{z}}/{\text{sp}}} \right)^{2} }} { }$ (8) $$\begin{aligned} {\text{Q}}_{{\text{p}}} & = 109.8 \times ({\text{WF}} \times {\text{EF}} \\ & \;\;\; \times {\text{SCF}} \times {{{\rm K}^{\prime}}}){ } \\ \end{aligned}$$ (9) $$\begin{aligned} {\text{sp}} & = 150.71 \times ({\text{WF}} \times {\text{EF}} \\ & \;\;\; \times {\text{SCF}} \times {{{\rm K}^{\prime}}})^{ - 0.3711} { } \\ \end{aligned}$$ (10) ${\text{SL}}_{{\text{r}}} = \frac{{2{\text{Z}}}}{{{\text{sr}}^{2} }} \times {\text{Q}}_{{\text{r}}} \times {\text{e}}^{{ - \left( {{\text{z}}/{\text{sr}}} \right)^{2} }}$ (11) $$\begin{aligned} {\text{Q}}_{{\text{r}}} & = 109.8 \times ({\text{WF}} \times {\text{EF}} \\ & \;\;\; \times {\text{SCF}} \times {{{\rm K}^{\prime}}} \times {\text{C}}){ } \\ \end{aligned}$$ (12) $$\begin{aligned} {\text{sr}} & = 150.71 \times ({\text{WF}} \times {\text{EF}} \\ & \;\;\; \times {\text{SCF}} \times {{{\rm K}^{\prime}}} \times {\text{C}})^{ - 0.3711} { } \\ \end{aligned}$$ (13)	${\text{F}}_{{\text{S}}} { }$ is the quantity of wind erosion control and sediment retention supply (kg/m^2^), ${\text{SL}}_{{\text{p}}} { }$ is potential wind erosion(kg/m^2^), ${\text{SL}}_{{\text{r}}} { }$ is the actual wind erosion(kg/m^2^),${\text{ Q}}_{{\text{p}}} { }$ is the maximum sediment transport capacity of potential wind power(kg/m),${\text{ sp }}$ is the actual critical plot length(m),${\text{ Q}}_{{\text{r}}} { }$ is the maximum sand transport capacity of the actual wind(kg/m),${\text{ sr }}$ is the potential critical plot length(m),${\text{ Z }}$ is the downwind distance, ${\text{WF }}$ is the climate factor, ${\text{EF}}$ and ${\text{SCF}}$ are the soil erodibility and soil crust factors respectively, ${{{\rm K}}}^{\prime }$ and ${\text{ C }}$ are the surface roughness and vegetation factors. respectively
	The actual wind erosion is used as the wind erosion control and sediment retention demand	${\text{F}}_{{\text{D}}} = \frac{{2{\text{Z}}}}{{{\text{sr}}^{2} }} \times {\text{Q}}_{{\text{r}}} \times {\text{e}}^{{ - \left( {{\text{z}}/{\text{sr}}} \right)^{2} }} { }$ (14)	${\text{F}}_{{\text{D}}} { }$ is the quantity of wind erosion control and sediment retention demand (kg/m^2^), other parameters are the same as those used in the equation

### Analysis of matching ESs supply and demand

#### Quantitative matching analysis

In this study, the ESs supply–demand ratio was used to link the actual supply of ESs to the human demand to quantitatively assess the matching relationship between the supply and demand of ESs^34^. The formula is as follows:

15 $${\text{ ESDR}} = \frac{{{\text{ESS}} - {\text{ESD}}}}{{({\text{ESS}}_{{{\text{max}}}} + {\text{ESD}}_{{{\text{max}}}} )/2}}$$

where ${\text{ESDR}}$ is the ratio of ecological supply to demand, and the ${\text{ESS}}$ and ${\text{ESD }}$ denote the actual supply and demand of ESs, respectively. ${\text{ ESS}}_{{{\text{max}}}} { }$ a and ${\text{ ESD}}_{{{\text{max}}}} { }$ represent the maximum supply and demand of ESs, respectively. ${\text{ESDR}}$ > 0 indicates that the supply of ESs exceeds the demand, ${\text{ESDR}}$ < 0 shows that supply is less than demand, and ${\text{ESDR}}$ = 0 denotes a balance between supply and demand.

#### Spatial matching analysis

This study used a four-quadrant model to explore the supply and demand patterns of each ESs. First, the supply and demand of ESs were standardized using the Z-score method^33^. Then, using the standardized supply as the X-axis and demand as the Y-axis, a two-dimensional coordinate system was constructed, and four matching modes were obtained and visualized, including high-high spatial matching, low-low spatial matching, low–high spatial misalignment and high-low spatial misalignment^38^.

### Integrated ESs supply–demand ratio and cold hotspot analysis

The integrated supply–demand ratio for ESs measures the state of overall horizontal ESs supply and demand, calculated as the arithmetic mean of ESs supply–demand ratio. The formula is as follows:

16 $${\text{CESDR}} = \frac{1}{{\text{n}}}\mathop \sum \limits_{{{\text{i}} = 1}}^{{\text{n}}} {\text{ESDR}}_{{\text{i}}}$$

where ${\text{ CESDR }}$ denotes the combined ecological supply–demand ratio.${\text{ n }}$ is the quantity of ESs, in this study ${\text{ n}} = 4$, and ${\text{ ESDR}}_{{\text{i }}}$ is the ecological supply–demand ratio for the ${\text{ i }}$ ESs.

Cold hotspot areas show the aggregation of high and low values, respectively. In this study, cold hotspots were used to explore the degree of distribution and aggregation of integrated ESs supply–demand ratio, and to identify areas of interest in ESs.

## Results

### Spatial–temporal evolution of ESs supply and demand

#### Spatial–temporal changes of ESs supply

From 2000 to 2020, the food supply, carbon sequestration, wind erosion control and sediment retention showed an increasing trend (Table 2). Among them, food supply increased from 32.69 to 77 t/km^2^, with a rapid growth rate; carbon sequestration increased from 8753.75 to 8882.19 t/hm^2^, with an increase of 1.47%; the supply of wind erosion control and sediment retention increased year by year, but the overall change is not large. However, the supply of water yield showed an “upward-downward” trend (Table 2), mainly because, from 2000 to 2010 the water area increased by 321.81 km^2^, and precipitation also increased by 22.92%, leading to an increase in water yield. In contrast, after 2010, the increase in water area shrank, and the significant increase in grassland area led to a reduction in runoff, which limited the water yield supply.

**Table 2:** Average and changes of ESs supply and demand in 2000–2020.

Type	Food supply (t/km^2^)		Carbon sequestration (t/hm^2^)		Water yield (m^3^/km^2^)		Wind erosion control and sediment retention (kg/m^2^)	
	Supply	Demand	Supply	Demand	Supply	Demand	Supply	Demand
2000	32.69	6.23	8753.75	0.40	521,788.98	32,032.52	1.61	28.69
2010	50.89	6.34	8855.41	0.51	691,674.71	30,125.92	1.67	28.37
2020	77	7.56	8882.19	0.72	536,334.92	29,035.27	1.75	27.50

**Figure 3:**
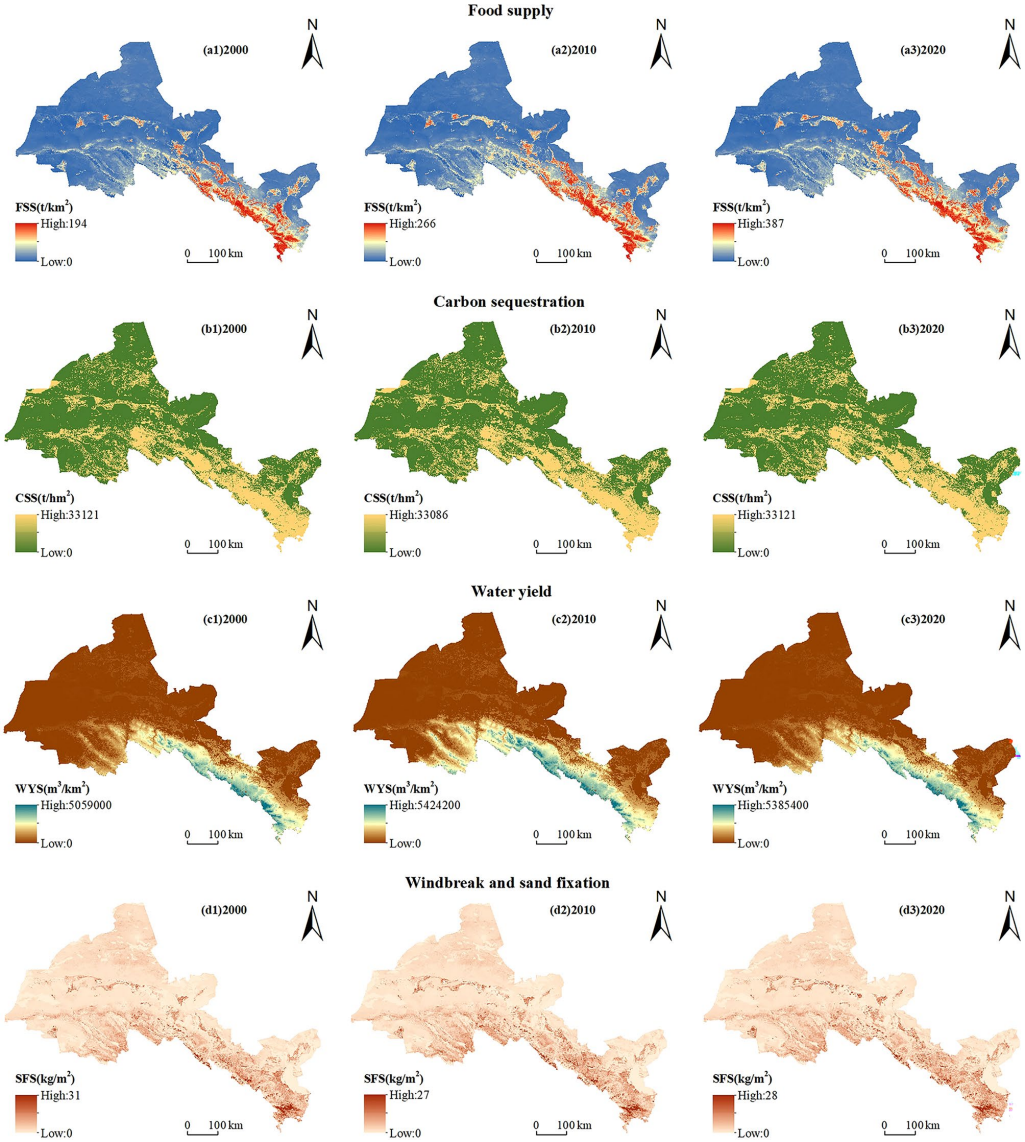
Spatial distribution and changes of ESs supply in Hexi Region from 2000 to 2020. (The map was generated by ArcGIS 10.8 http://www.esri.com/software/arcgis and does not require any permission from anywhere).

Spatially, there is little spatial variation in the supply of ESs in different periods, but there is some spatial variation in the supply of different ESs, and this spatial variation exists across ESs (Fig. 3). Specifically, the high-value areas of food supply are mainly distributed in the Qilian Mountains and Oasis Area, mainly because these areas are dominated by forests, grasslands, water bodies, and cultivated land, and the production of foods, aquatic products, and livestock products is high (Fig. 3a1–a3). The supply of carbon sequestration shows a spatial pattern of “high in the southeast and low in the northwest”. Qilian Mountains and Oasis Area are high-value areas for carbon sequestration. These areas have a good ecological environment and high carbon density, which have a positive impact on carbon sequestration (Fig. 3b1–b3). The high-value area of water yield supply is mainly distributed in the Qilian Mountains, and its spatial distribution is mainly influenced by annual precipitation and land use types (Fig. 3c1–c3). The supply of wind erosion control and sediment retention also has a spatial pattern of “high in the southeast and low in the northwest”. The high-value areas are mainly concentrated in the Qilian Mountains and Oasis Area. These areas are mainly forest, grassland, and cropland, with relatively high vegetation cover, small soil particle size, and good texture, and the potential wind erosion of the soil is greater than the actual wind erosion (Fig. 3d1–d3).

#### Spatial-temporal changes of ESs demand

From 2000 to 2020, the average demand of food supply and carbon sequestration increased annually, while the average water yield demand and wind erosion control and sediment retention demand decreased. Among them, the demand for food supply increased by 1.33%, which was due to the increase of 0.054t in human demand for food, aquatic products, and livestock products. The 32% increase in demand for carbon sequestration is due to the increase in fossil fuel consumption and the resulting 1.6t increase in per capita carbon emissions over the past 20 years, as economic development and urbanization levels have risen. The demand for water yield decreased by − 9.36%. With the improvement of production and domestic water efficiency, the total water consumption for agriculture, industry, domestic water, and ecology decreased by 7.97 × 10^8^ m^3^, which made the demand for water yield decrease. Demand for wind erosion control and sediment retention decreased by − 4.15%. Due to the implementation of ecological policies such as “Returning Farmland to Forest and Grassland”, “Three North Shelterbelt Construction”, “Comprehensive River Basin Management” and “Sand Prevention and Control Project”, the actual wind erosion decreased (Table 2).

**Figure 4:**
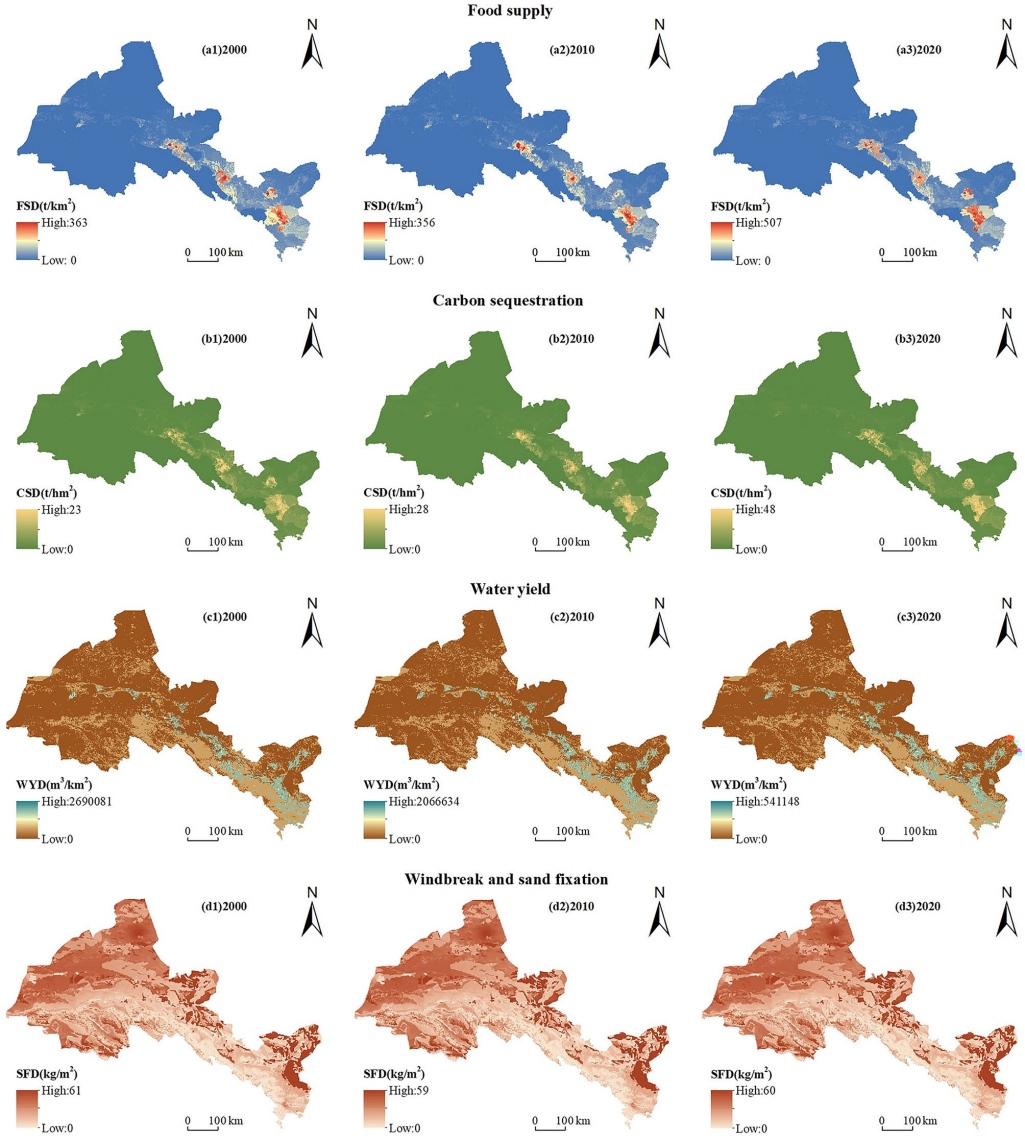
Spatial distribution and changes of ESs demand in Hexi Region from 2000 to 2020. (The map was generated by ArcGIS 10.8 http://www.esri.com/software/arcgis and does not require any permission from anywhere).

Spatially, there are significant spatial differences in the demand for ESs across ecosystems. Areas with high demand for food supply are concentrated in Jiayuguan, Suzhou, Ganzhou, Jinchuan, and Liangzhou districts in the Oasis Area, where the average annual food demand is as high as 47.28 t/km^2^ and these areas have high population density, high urbanization, high living standards and high consumption expenditure on various food items. Low-value areas include Sunan and Aksai in the Qilian Mountains and Subei in the Desert Area. These areas are vast and sparsely populated, and food consumption is less than 0.35 t/km^2^. The remaining areas are in the middle range, with a food demand of 5.92 t/km^2^ (Fig. 4a1–a3). The high-value areas of carbon sequestration demand are also distributed in Jiayuguan City, Suzhou District, Ganzhou District, Jinchuan District, and Liangzhou District in the Oasis Area, with an average annual demand of 3.56 t/hm^2^. Due to the dense population and high level of industrial and service development, these areas have high fossil fuel consumption and carbon emissions. However, the average annual carbon sequestration demand in other areas is less than 0.5 t/hm^2^ (Fig. 4b1–b3). The high-value areas of water yield demand are concentrated in the Oasis Area, and the annual water demand is as high as 10,328.63 m^3^/km^2^. These areas include the agricultural areas and the built-up areas in the Hexi area. The agricultural areas are mainly cultivated land, and agricultural irrigation water accounts for about 80% of the total water consumption. The built-up areas are densely populated, industrially developed and have large industrial and domestic water consumption. The remaining areas are dominated by forests, grasslands, water bodies and unused land. The ecological water consumption of forests, grasslands and water bodies is only 10%. Unused land is not suitable for industrial and agricultural production and human life, and the annual water production demand is less than 180 m^3^/km^2^ (Fig. 4c1–c3). The areas with high demand for wind erosion control and sediment retention are mainly distributed in the desert areas affected by the Tengger Desert and Badain Jaran Desert, with poor soil texture, large particle size, low vegetation cover, and severe wind erosion. The low-value areas of demand for wind erosion control and sediment retention are mainly distributed in Qilian Mountains and Oasis Area, where the ecological environment is good, soil texture is excellent and wind erosion is less (Fig. 4d1–d3).

### Supply-demand matching characteristics of ESs

#### Quantitative matching characteristics

From 2000 to 2020, the supply–demand ratios of food supply, carbon sequestration and water yield in the Hexi region were all greater than 0, indicating a state of oversupply, while the supply–demand ratios of wind erosion control and sediment retention were always less than 0, indicating a state of undersupply. Spatially, there are obvious spatial differences in the quantity matching between the supply and demand of each ESs.

**Figure 5:**
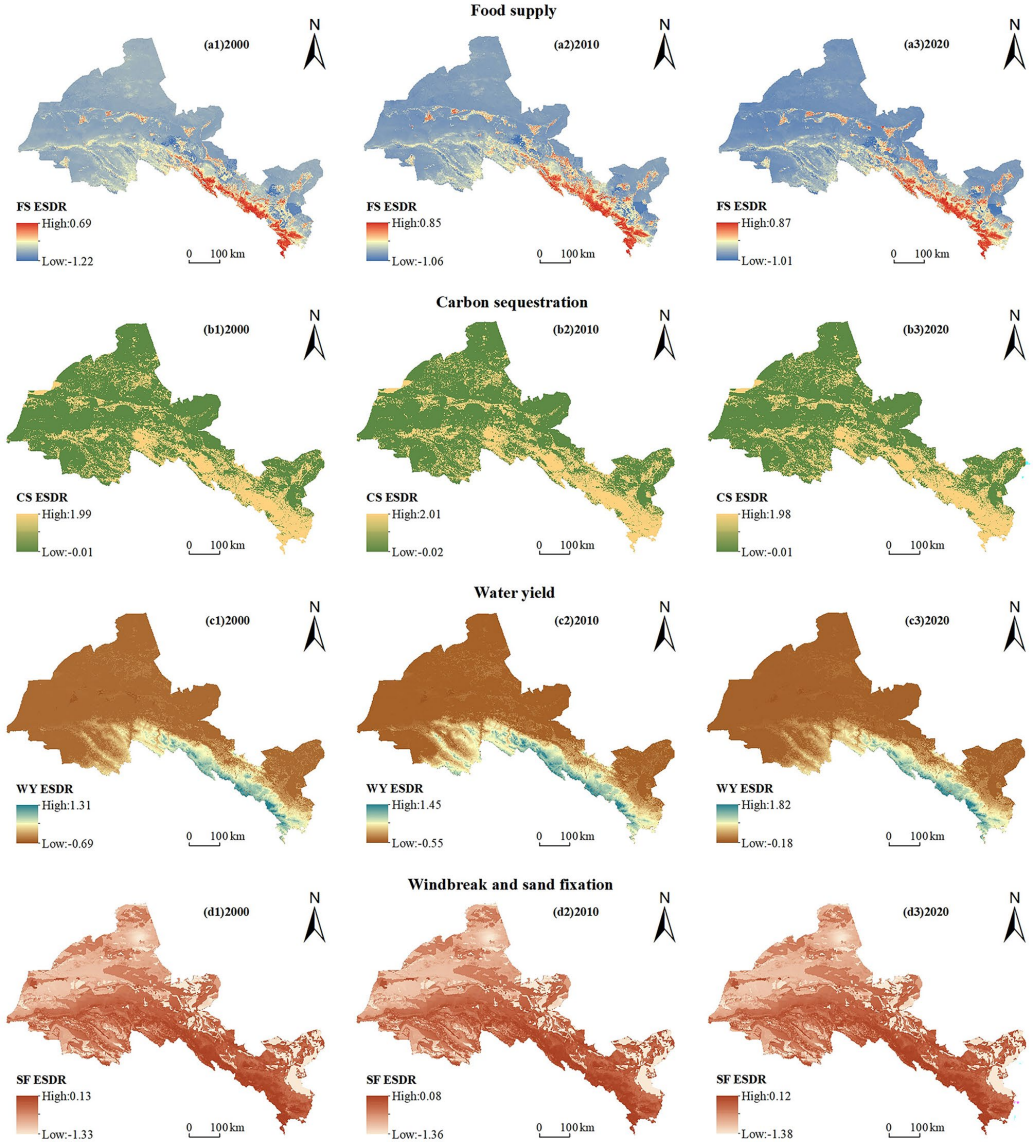
Spatial pattern of supply–demand ratio of various ESs in Hexi Region from 2000 to 2020. (The map was generated by ArcGIS 10.8 http://www.esri.com/software/arcgis and does not require any permission from anywhere).

From 2000 to 2020, the supply–demand ratio of food supply increased, with supply exceeding demand on average (Table 3). Regarding spatial distribution, the food supply is in a state of oversupply in the Qilian Mountains and Oasis Area, which gradually expands over time, while the other regions are in a state of short supply (Fig. 5a1–a3). The supply–demand ratios of carbon sequestration did not increase significantly, with supply exceeding demand on average (Table 3). From the spatial distribution, the positive values of ESs supply–demand ratio are mainly distributed in the Qilian Mountains with high forest and grass coverage, followed by the Oasis Area with abundant arable land resources, and the negative values are mainly concentrated in the Desert Area (Fig. 5b1–b3). The supply–demand ratios of water yield tend to decline with the changes, and it has been in a situation of oversupply (Table 3). In the Qilian Mountains and surrounding areas, there is a spatial surplus of water yield supply compared to demand, and the distribution is banded along the Qilian Mountains. Additionally, there is a deficit in other regions (Fig. 5c1–c3). The supply–demand ratio of wind erosion control and sediment retention is always less than 0, which is in a state of short supply (Table 3). The reason for this phenomenon is the low vegetation cover in the Hexi region, the existence of a large area of the Gobi Desert, the bare surface, and some areas on the edge of the desert, the actual wind erosion is large. The long-term imbalance has led to strong winds and a lot of sand in the area, making the ecological environment fragile. At the same time, regions near the desert also face the risk of being buried by the desert. However, a number of ecological restoration projects have been undertaken in the region, which have reduced the imbalance between supply and demand for regional wind erosion control and sediment retention over the years (Fig. 5d1–d3).

**Table 3:** Average and changes of ESs supply–demand matching in 2000–2020.

	Food supply (t/km^2^)	Carbon sequestration (t/hm^2^)	Water yield (m^3^/km^2^)	Wind erosion control and sediment retention (kg/m^2^)
2000	0.095	0.528	0.126	− 0.594
2010	0.143	0.534	0.177	− 0.605
2020	0.156	0.536	0.171	− 0.614

#### Spatial matching characteristics

From 2000 to 2020, the spatial matching of food supply, carbon sequestration, water yield, wind erosion control and sediment retention in the Hexi region was dominated by low-low spatial matching zones, which were mainly distributed in the north-west of the region. The Gobi Desert was widely distributed in these areas and the natural environment was harsh, resulting in low supply and demand of its ESs.

**Figure 6:**
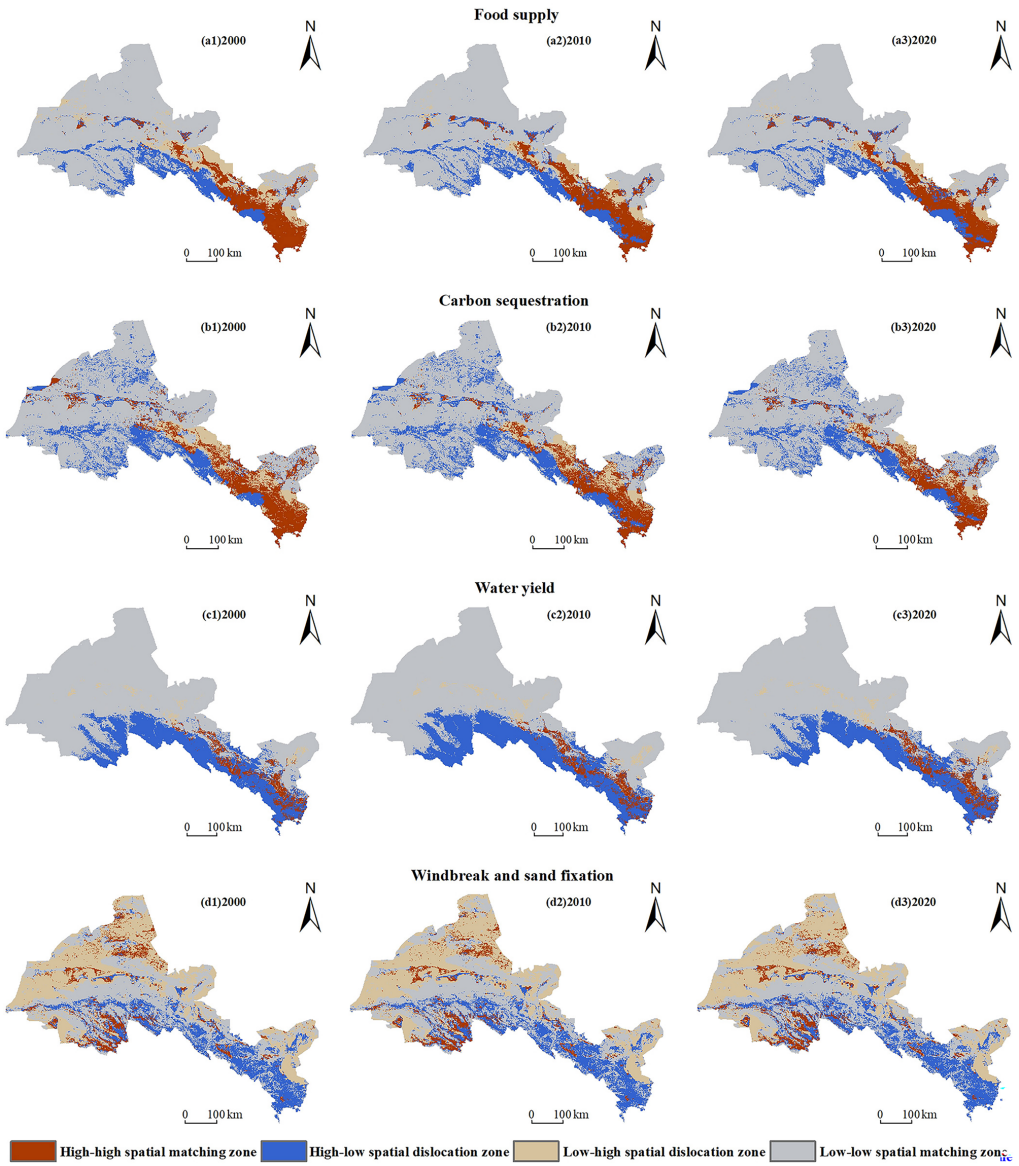
Spatial matching relationships of supply and demand of ESs in Hexi Region from 2000 to 2020. (The map was generated by ArcGIS 10.8 http://www.esri.com/software/arcgis and does not require any permission from anywhere).

The food supply is mainly based on the low-low spatial matching zones. It is because the proportion of unused land in the Hexi region is as high as 67.24%, the NDVI value of unused land is low, the vegetation growth is poor, the food supply is low, and the population is sparse, so the demand for food supply is low. Among them, the high-high spatial matching zone is mainly distributed in the Oasis Area such as Shandan, Yongchang and Tianzhu, with the area ratio decreasing by 0.41%; the high-low spatial dislocation zone is mainly located in the southern Qilian Mountains and the edge of the Oasis Area, with the area ratio decreasing by 2.45%; the low–high spatial dislocation zone is mainly concentrated in Linze, Ganzhou, Yongchang, Liangzhou, the northern part of Gulang and most of Jiayuguan, Suzhou, Linze and Jinchuan in the Oasis Area, with the area ratio decreasing by 4.21%; the low-low spatial matching zone is mainly located in the northwestern Desert Area and the edge of Minqin Oasis, with the area ratio increasing by 4.21% (Fig. 6a1–a3). Carbon sequestration is mainly in low-low spatial matching areas, followed by high-low spatial dislocation areas. Among them, the high-high spatial matching is mainly concentrated in the Oasis Area, with the area ratio decreasing by 1.98%; the high-low spatial dislocation zone is primarily distributed in the Southern Qilian Mountains and part of the areas in the northwest, with the area ratio increasing by 2.66%; and low–high spatial dislocation zone is primarily concentrated in Jiayuguan, Jinchang, Suzhou, Gaotai, and Liangzhou in the Oasis Area; the low-low spatial matching zone is mainly distributed in the northwestern Gobi Desert and the edge of Minqin Oasis, the increase is not large (Fig. 6b1–b3). The water yield is mainly focused on low-low spatial matching areas, followed by high-low spatial dislocation areas. Among them, the high-high spatial matching zone is concentrated in the central-eastern Oasis Area, accounting for an increase of 0.31%; the high-low spatial dislocation zone is distributed in the Qilian Mountains, accounting for a decrease of 0.95%; the low–high spatial dislocation zone is scattered, with a proportion less than 5%; the low-low spatial matching zone is mainly distributed in the northwestern Desert Area and the edge of Minqin Oasis, the decline is small (Fig. 6c1–c3). The wind erosion control and sediment retention is mainly in low-low spatial matching areas, followed by low–high spatial dislocation areas. Among them, the high-high spatial matching zone is scattered and occupies a relatively low area; the high-low spatial dislocation zone is mainly distributed in the southern Qilian Mountains and Oasis Area. with an increase in area of 0.76%; the low–high spatial dislocation zone is distributed in the northwest Gobi Desert, the edge of Jinta and Minqin Oasis, with an increase in area of 0.22%; the low-low spatial matching zone is mainly concentrated in the northwest Desert Area, while the rest of regions are scattered and small (Fig. 6d1–d3).

### Integrated ESs supply–demand ratio and cold hotspot analysis

**Figure 7:**
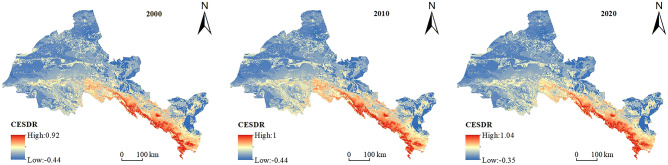
Spatial distribution of supply–demand ratio of integrated ESs in Hexi Region. (The map was generated by ArcGIS 10.8 http://www.esri.com/software/arcgis and does not require any permission from anywhere).

**Figure 8:**
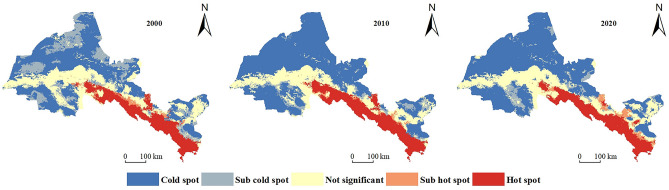
Distribution of cold and hot spots in Hexi region from 2000 to 2020. (The map was generated by ArcGIS 10.8 http://www.esri.com/software/arcgis and does not require any permission from anywhere).

In the Hexi region, the integrated ESs supply–demand ratio has an average value of 0.039, 0.062, and 0.061 in 2000, 2010, and 2020, respectively. The integrated ESs supply–demand ratio fluctuates, but generally increases, indicating that the ecosystem is gradually becoming more balanced. The southern Qilian Mountains and the central oasis agricultural areas, which have high levels of forest and grass cover, rich agricultural resources, and substantially improved ecological environment quality, are the primary locations of the high-value regions of integrated ESs supply–demand ratio (Fig. 7). From 2000 to 2020, the proportion of cold spot areas increased from 42.16 to 54.88%, while the proportion of sub-cold spot areas decreased from 17.53 to 6.46%, and the total balance of cold spots and sub-cold spots increased from 59.69 to 61.34%. The majority of the northwest region, as well as Minqin, Liangzhou, and Gulang, are the main locations for it (Fig. 8). Cold spots and sub-cold spots occupy more than half of the region and are concentrated in northwest of the region, indicating that integrated ESs supply–demand ratio is seriously unbalanced and low-value aggregation is obvious in the northwest of the region. The proportion of hot pot areas decreased from 14.16 to 13.28%, while the proportion of sub-hot pot areas increased from 2.18 to 6.46%, and the total proportion of hot pots and sub-hot pots increased from 16.34 to 16.36%. It is primarily found in the southern Qilian Mountains and a few Oasis Area. The Oasis Area is where the phenomenon of spreading to the periphery first appeared (Fig. 8), indicating that the supply and demand of ESs are more balanced in the southern Qilian Mountains. In some areas of the Oasis Area, the supply–demand ratio is approaching equilibrium due to advancements in agricultural technology.

## Discussion and analysis

### Rationality of ESs supply and demand assessment

Assessment of ESs supply and demand is a major aspect of ESs research. The assessment matrix and value equivalence methods for ESs supply and demand can to some extent meet current research needs, but these semi-quantitative methods are subject to strong subjectivity and uncertainty, and cannot completely depict the spatial pattern of ESs supply and demand in small areas^15^. For large-scale ESs supply and demand assessment, material quality assessment can better reflect the actual supply and demand situation in the study area than value assessment^15,33^. In this study, the actual material quality of specific ESs is used to measure the supply and demand of each ESs to reflect the actual situation of ESs in the study area^39^. We chose the InVEST model and RWEQ to assess the supply of ESs. These methods are relatively mature and have certain advantages in spatial visualization and dynamic research. Research on demand indicators has been sparse and lacking in relation to the quantification of ESs demand up to this point, primarily as a result of the dearth of data support and a theoretical framework for supply–demand balance analysis^9^. Some scholars have also used population density, per capita GDP, and land use intensity to assess the demand for ESs comprehensively. However, such methods do not consider the specific needs of different ESs^7^. In this study, human consumption and actual wind erosion were selected as demand indicators based on the specificity of each ESs type, where human demand included food use, carbon emissions and water consumption to evaluate the actual demand for each ESs^39^.

The accuracy of the research results is improved by the method of material quality evaluation. We find that there is a certain spatial distribution law for ESs supply. Among them, Qilian Mountains is a high-value area of ESs, Oasis Area is a high-value area of food supply, carbon sequestration, and wind erosion control and sediment retention, and Desert Area is a low-value area of ESs. The spatial distribution of ESs supply is consistent with the ecosystem pattern, which further verifies the accuracy of the quality assessment methods such as the InVEST model and RWEQ. In addition, the demand for ESs also has a certain spatial distribution law. Among them, the high-value areas of food supply and carbon sequestration demand are mainly concentrated in Jiayuguan City, Suzhou District, Ganzhou District, Jinchuan District and Liangzhou District of Oasis Area, which is consistent with the spatial distribution of population density and the spatial agglomeration characteristics of human activities. The high-value area of water yield demand is concentrated in Oasis Area, which accords with the actual situation of large water consumption in Oasis Area. The areas with high demand for wind erosion control and sediment retention are mainly distributed in Desert Area, which is closely related to the harsh natural environment and also meets the actual demand for wind erosion control and sediment retention in the Desert Area. These results are in line with the actual demand of ESs in the Hexi region, and also illustrate the importance of evaluating the demand of specific ESs.

### Matching supply and demand for ESs and policy recommendations

Influenced by many factors, such as the spatial difference of ecosystem, the level of socio-economic development and regional ecological policies, the relationship between supply and demand of ecosystem services is dynamic, and there is a general spatial mismatch between supply and demand of ESs^20,33^, mainly including the mismatch in quantity and space. In this study, with reference to the relevant literature and in light of the actual situation in the Hexi region, we explored the matching relationship between the supply and demand of four ESs, namely food supply, carbon sequestration, water yield, wind erosion control and sediment retention, at both the quantitative and spatial levels. Future studies may also attempt to explore the matching relationship between the supply and demand of maintenance services and cultural services related to the human environment^15,33^.

We introduce the supply–demand ratio of ESs to measure the quantitative relationship between the supply and demand of ESs. It is found that the supply–demand ratio of ESs is in a state of oversupply in the Qilian Mountains and in a state of shortage in the Desert Area. The supply–demand ratio of food supply, carbon sequestration, and wind erosion control and sediment retention is in a state of oversupply in the Oasis Area, while the supply–demand ratio of water yield is in a state of shortage in the Oasis Area. In addition, the matching patterns of supply–demand of four ESs in space were evaluated through quadrant differentiation. We found that ESs are mainly low-low spatial matching areas, and are concentrated in the Desert Areas. Therefore, we should protect the ecological environment in the southern Qilian Mountains, strengthen regional ecological management, and maintain the integrity of the ecosystem^40,41^. For the core area of Qilian Mountains, we should strengthen the protection of the Qilian Mountains glacier and water conservancy ecological function area in the future. Mining and grazing activities in the area must be subordinated to the protection of the water conservancy function, and at the same time, the construction of national parks should be strengthened. For the border area of Qilian Mountains, we can gradually reduce the interference intensity of animal husbandry and human activities in mountainous areas through measures such as livestock fencing with grass and ecological migration in mountainous areas, and also strictly control the scale of development to prevent the ecological space from being squeezed. The central Oasis Area is the essence and hub of the Hexi region, so the protection of the oasis should be strengthened^42^. The urban districts in the Oasis Area are mainly industrial and service districts, so it is necessary to promote the transformation of industrial structure, promote the development of green and energy-saving industries, and reduce the pressure on the system caused by industrial activities. The remaining counties in the Oasis Area are mainly agricultural activity, and these areas should focus on the development of water-saving, efficient, and intensive sustainable agriculture and take the improvement of irrigation efficiency, the control of secondary soil salinization, and the construction of efficient water-saving agricultural system as important and long-term tasks. With regard to the development of the Desert Area, it is necessary to carry out relevant ecological restoration projects and strengthen regional ecological environmental management in the future so as to enhance the ability of wind erosion control and sediment retention^42^. In addition, it is also necessary to reduce the interference of unreasonable human activities on the system, promote the transformation of farmers' livelihood and industrial structure, and enhance the regional development potential^15^. We suggest that policymakers should continue to implement ecological restoration projects, build an ecological protection system focusing on wind erosion control and sediment retention, strengthen the management of degraded grasslands, and enhance the ability of regional sand prevention and control. In general, the development focus of the Hexi region should to be “contain water in the south, protect oasis in the middle, and prevent wind and sand in the north”. In the future, it is necessary to optimize the main ESs functions of each region and establish an inter-regional ecological compensation mechanism to ensure the balance between the supply and demand of regional ESs, so as to promote the sustainable development of the Hexi region.

### Relationship between integrated ESs supply–demand ratio and individual ESs supply–demand ratio

The supply–demand ratio of comprehensive ESs is the result of the combined effects of the supply–demand ratios of various ESs, and the two are interconnected and interdependent. From 2000 to 2020, the supply–demand ratios of food supply, carbon sequestration and water yield were all greater than 0 and increasing. These ESs are in a state of oversupply in terms of quantity, and their supply–demand ratios play a positive role in promoting the supply–demand ratio of integrated ESs, while at the same time contributing to the state of oversupply of total ESs, which can promote the sustainable development of the ecosystem. However, the supply–demand ratio of wind erosion control and sediment retention has always been less than 0, and the quantity adjustment is in a state of shortage. Its supply–demand ratio is negative for improving the supply–demand ratio of integrated ESs, and it also hinders the healthy development of the ecosystem. Over time, the negative impact of the supply–demand ratio of wind erosion control and sediment retention on the supply–demand ratio of integrated ESs has been decreasing year by year, which is related to the implementation of a number of ecological restoration projects that have reduced regional ecological risks and played a positive role in regional ecological security^27,28,31^. Spatially, the supply–demand ratio of integrated ESs is basically the same as that of each ESs. The area where supply exceeds demand is mainly concentrated in Qilian Mountain, which have a high degree of vegetation cover and better ecological environmental quality. At the same time, due to the constraints of topography and other factors, the population density in the area is low, and social and economic activities have little interference with the local ecological environment, forming a state where supply exceeds demand^43^.

## Conclusions

This study quantified the supply and demand of four ESs based on spatial analysis tools such as the InVEST model and ArcGIS. The study identified the quantitative match and spatial match types of ESs supply and demand, and analyzed the integrated ESs supply–demand ratio and their hot and cold spots. The results of the study are as follows:

1. From the perspective of supply, the overall supply of each ESs in the Hexi region shows an increasing trend from 2000 to 2020, with the food supply service having the fastest growth rate, exceeding 50%. The spatial distribution pattern of each ESs supply is “high in the southeast and low in the northwest”. From a demand perspective, the demand for food supply and carbon sequestration services has shown an increasing trend from 2000 to 2020. In particular, the increase in demand for carbon sequestration services has exceeded 30%, while the demand for water production and wind erosion control and sediment retention services has shown a decreasing trend. The spatial distribution of food supply and carbon sequestration services is consistent with population density, with high-value areas mainly concentrated in urban and peripheral areas. The high-value areas for water production services are mainly distributed in oasis agricultural areas, while the high-value areas for wind erosion control and sediment retention services are concentrated in desert and neighboring areas.
2. In terms of quantity matching, the food supply, carbon sequestration, and water yield in the Hexi region are all on average in a state of oversupply, with the oversupply mainly concentrated in the southern Qilian Mountains area, followed by the central oasis plain area. The quantitative match for wind erosion control and sediment retention have always been in short supply, and their spatial distribution is basically consistent with that of other ESs. From the perspective of spatial matching, all ESs are dominated by low-low spatial matching areas, with large areas widely concentrated in the northwest desert region.
3. From 2000 to 2020, the supply–demand ratio of comprehensive ESs in the Hexi region exhibited a fluctuating trend, but a general increase was observed. The total proportion of cold and sub-cold spots increased from 59.69% to 61.34%, covering more than half of the regional area and concentrated in the northwest. The total proportion of hot and sub-hot spots increased from 16.34% to 16.36%, mainly appearing in the southern Qilian Mountain area and a few oasis areas, with a phenomenon of spreading to the surrounding areas observed in the oasis area.

## Data Availability

The original contributions presented in the study are included in the article, further inquiries can be directed to the corresponding authors.
